# Poor prognostic clinicopathologic features correlate with VEGF expression but not with PTEN expression in squamous cell carcinoma of the larynx

**DOI:** 10.1186/1746-1596-5-35

**Published:** 2010-06-14

**Authors:** Yurdanur Sullu, Seda Gun, Sinan Atmaca, Filiz Karagoz, Bedri Kandemir

**Affiliations:** 1 Ondokuz Mayis University, Faculty of Medicine, Department of Pathology, Samsun, Turkey; 2 Mehmet Aydin Education and Research Hospital, Department of Pathology, Samsun, Turkey; 3 Ondokuz Mayis University, Faculty of Medicine, Department of Ear- Nose- and Throat Disease, Head and Neck Surgery, Samsun, Turkey

## Abstract

**Background:**

The aim of this study was to assess the relationship between expression of vascular endothelial growth factor (VEGF) and phosphatase and tensin homolog deleted in chromosome ten (PTEN), angiogenesis and clinicopathological parameters of squamous cell carcinoma of the larynx.

**Methods:**

We examined immunohistochemical expression of VEGF and PTEN and CD34 for microvessel density (MVD) in sections of formalin-fixed, paraffin embedded tissue blocks of 140 patients with squamous cell carcinoma of the larynx. The intensity of VEGF and PTEN staining and the proportion of cells staining were scored.

**Results:**

The tumor grade was not significantly related to PTEN expression, but it was to VEGF expression (p = 0.400; p = 0.015, respectively). While there was no significant relationship between PTEN expression and tumor size and cartilage invasion (p = 0.311, p = 0.128), there was a significant relationship between the severity of VEGF expression and tumor size (p = 0.006) and lymph node metastasis (p = 0.048) but not cartilage invasion (p = 0.129). MVD was significantly higher in high-grade tumors (p = 0.003) but had no significant relationship between MVD, lymph node metastasis, and cartilage invasion (p = 0.815, p = 0.204). There was also no significant relationship between PTEN and VEGF expression (p = 0.161) and between PTEN and VEGF expression and the MVD (p = 0.120 and p = 0.175, respectively).

**Conclusions:**

Increased VEGF expression may play an important role in the outcome of squamous cell carcinoma of the larynx. PTEN expression was not related to VEGF expression and clinicopathological features of squamous cell carcinoma of the larynx.

## Introduction

Although tumor development is a multi-stage process, angiogenesis is essential to tumor growth and metastasis. Angiogenesis is regulated by the balance between positive and negative regulatory molecules released by tumor cells and other cells in the environment. The most important molecules positively affecting angiogenesis are basic fibroblast growth factor, vascular endothelial growth factor (VEGF), interleukin-8 platelet-derived growth factor, and hepatocyte growth factor. Thrombospondin 1, platelet factor-4, angiostatin, endostatin, IFN-α, and metalloproteinase tissue inhibitors are inhibitors of angiogenesis [1]. The VEGF gene is located on the sixth chromosome. VEGF is a heparin-binding glycoprotein with at least four molecular forms. It enhances vascular permeability and induces endothelial cell growth, proliferation, migration and differentiation [2]. Phosphatase and tensin homolog deleted in chromosome ten (PTEN) is a tumor-suppressor gene located on the tenth chromosome that has a role in the progression of the cell cycle and apoptosis. It has been reported that PTEN inhibits angiogenesis by inactivating the phosphatidilinositol 3-kinase (PI3K) signal pathway to reduce VEGF expression [3].

The prognostic value of angiogenesis has been reported in various tumors such as breast, gastric and ovary cancer [4-6]. The results of studies on VEGF expression and tumor angiogenesis and the relationship between the clinicopathological factors and prognosis in head and neck tumors, particularly in squamous cell carcinoma of the larynx, are controversial [7,8]. The effect of VEGF expression and angiogenesis on the clinicopathological parameters and prognosis has become more relevant because of the use of angiogenesis inhibitors in cancer treatment [9].

The aim of this study was to assess the relationship between VEGF and PTEN expression and angiogenesis, tumor differentiation, invasion and lymph node metastasis in squamous cell carcinoma of the larynx.

## Materials and methods

Sections from laryngectomy specimens from 140 patients who were diagnosed with squamous cell cancer between 1989 and 2007 in Ondokuz Mayis University Faculty of Medicine, Department of Pathology, were re-examined and grading was performed according to WHO as well (grade I), moderately ( grade II) and poorly (grade III) differentiated [10]. Four micron sections were obtained from the convenient blocks and immunohistochemical studies were performed using PTEN (monoclonal mouse Ab, clone 28H6, Neomarkers, CA, USA), VEGF (identifying all VEGF types, polyclonal rabbit Ab, IgG, Neomarkers, CA, USA) and CD34 (monoclonal mouse Ab-1, clone QBEnd/10) primary antibodies using the streptavidin biotin peroxidase method. The best dilution rates were recorded by various attempts. VEGF, PTEN and CD 34 were diluted at 1/100. The endogenous peroxidase activity of deparaffinized sections was eliminated by incubation for 10 min in 3% hydrogen peroxide solution. Then the sections were boiled for 35 min in citrate buffer and left to cool down for 20 min. Block antibodies were applied to the sections for 5 min and VEGF, PTEN and CD34 primary antibodies each were applied for 1 h. The sections were then incubated with biotinylated anti-immunglobulin and streptavidin-peroxidase conjugate for 10 min each. A kit containing 3,3'diaminobenzidin (DAB) (Dako, Carpinterra,, USA) was used as the coloring agent. Finally, the sections were stained with Mayer's Hematoxylin for 60 min. Until DAB application, PH 7,6 phosphate buffers were used in all stages, while distilled water was used following DAB application. The procedures were performed at room temperature. As positive controls, we used an angiosarcoma section for VEGF and CD34, prostatic adenocarcinoma for PTEN. Negative control slides were treated without the primary antibody. PTEN expression was assessed by nuclear staining, and VEGF and CD34 expressions were assessed by cytoplasmic staining. The extent and severity of staining were assessed semi-quantitatively for PTEN and VEGF. The extent of staining was classified as 0 if 0-10% of tumor cells were stained, 1 if 11-25% of tumor cells were stained, 2 if 26-50% were stained, and staining 3 if more than 50% were stained.

The severity of staining was classified as 1 if it was light yellow, 2 if it was dark yellow, and 3 if it was brown. The sum of these two classifications was scored as negative (0) if the score was 0-2, mild (1) if the score was 3-4, and severe if the score were 5-6. The density of micro-vessels was calculated by averaging the number of micro-vessels counted in four areas with intense vessels around the tumor in ×200 magnification after visualization by ×100 magnification of CD34-stained sections [11].

Statistical analysis was performed using the "SPSS 12.0 for Windows" program. Data were expressed as mean ± standard deviation. Sommer's D statistics were used to evaluate the directional relation between tumor grade and PTEN and VEGF expression. The Chi-square and Fisher's exact test were used to compare the expression scores of PTEN and VEGF between MVD, tumor size, lymph node metastasis and cartilage invasion. In this test, negative and mild positive cells were combined because of less than 5 number cases. ANOVA test was used to evaluate the relation between MVD and tumor grade, expression scores of VEGF and PTEN. The level of significance was set as p < 0.05.

## Results

Of the 140 cases, 3 were females and 137 were males. The mean age was 58 ± 9.8 (range = 35-97) years. Of the cases, 76 had grade 1 squamous cell carcinoma, 41 had grade 2 and 23 had grade 3 squamous cell carcinoma. There was no lymph node metastasis in 91 cases and there was lymph node metastasis in 49 cases. The cartilage invasion was positive in 71 and negative in 69 cases.

**Figure 1:**
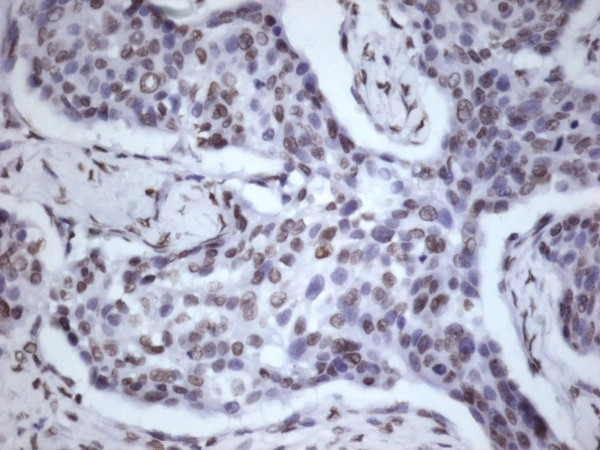
**Immunohistochemical demonstration of PTEN expression in grade 2 laryngeal squamous cell carcinoma, (severe, score 6) ×400**.

**Figure 2:**
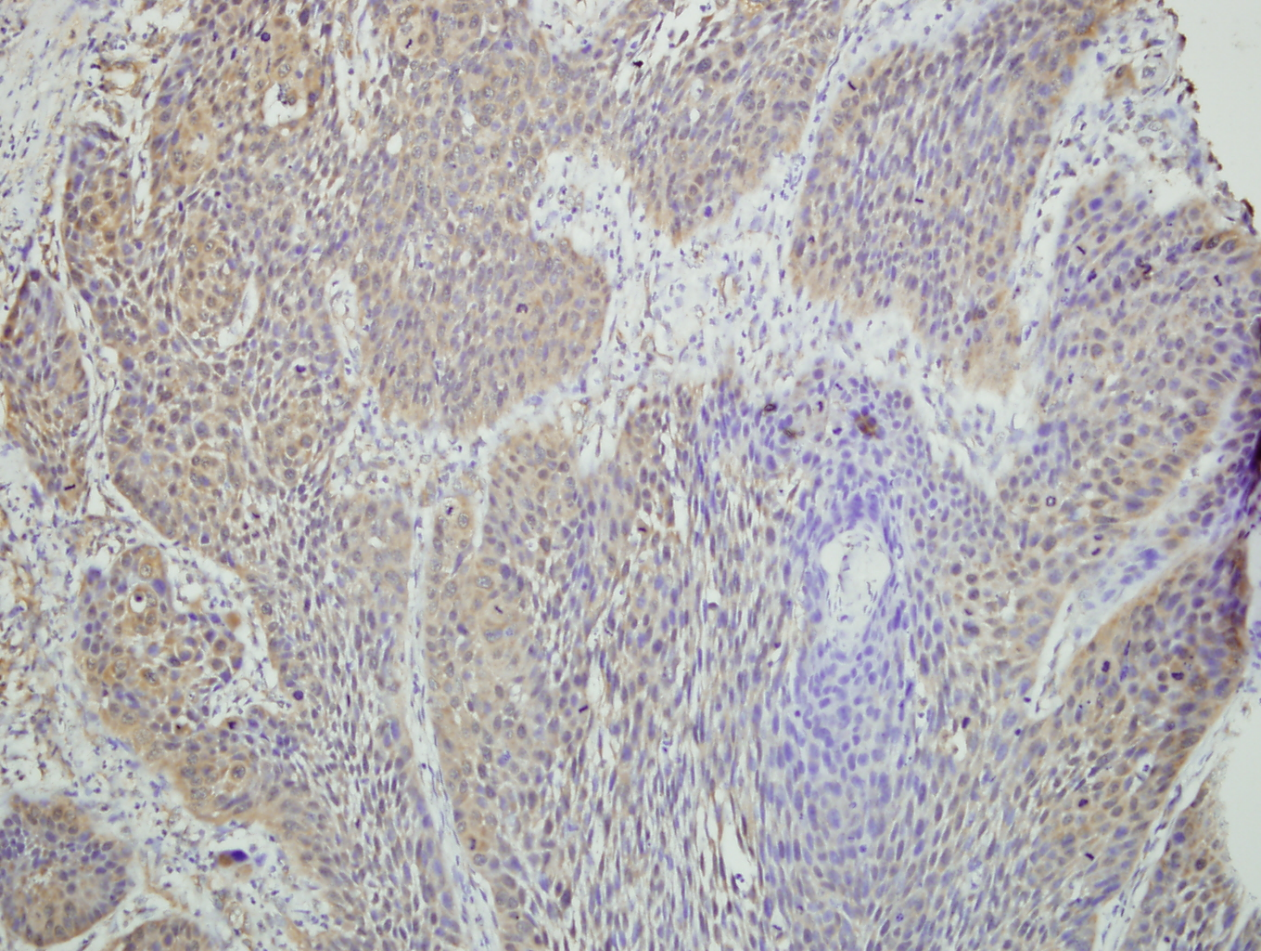
**Immunohistochemical demonstration of VEGF expression in grade 2 laryngeal squamous cell carcinoma, (mild, score 4) ×200**.

PTEN expression was negative in 26 cases (18.6%), mild positive in 43 (30.7%), and severe positive in 71 (50.7%) cases. VEGF expression was negative in 10 (7.1%) cases, mild positive in 28 (20.0%), and severe positive in 102 (72.9%) cases. The tumor grade was not significantly related to PTEN expression, but it was significantly related to VEGF expression (p = 0.400; p = 0.015, respectively) (Table 1, Figure 1 and 2).

**Table 1:** Association of VEGF, PTEN expression and MVD with tumor grade in laryngeal carcinoma (mean ± standard error of mean).

		PTEN				VEGF					
							
	N(%)	Negative	Mild	Severe	P	Negative	Mild	Severe	P	MVD*	P
Grade					0.400				0.015		0.003
Grade 1	76	11(14)	27(36)	38(50)		7(9)	18(24)	51(67)		43.0 ± 2.6	
Grade 2	41	6(15)	11(27)	24(58)		2(5)	10(24)	29(71)		40.4 ± 2.8	
Grade 3	23	9(39)	5(22)	9(39)		1(4)		22(96)		59.5 ± 5.8	

Total (%)	140	26(18)	43(31)	71(51)		10(7)	28(20)	102(73)			

**Figure 3:**
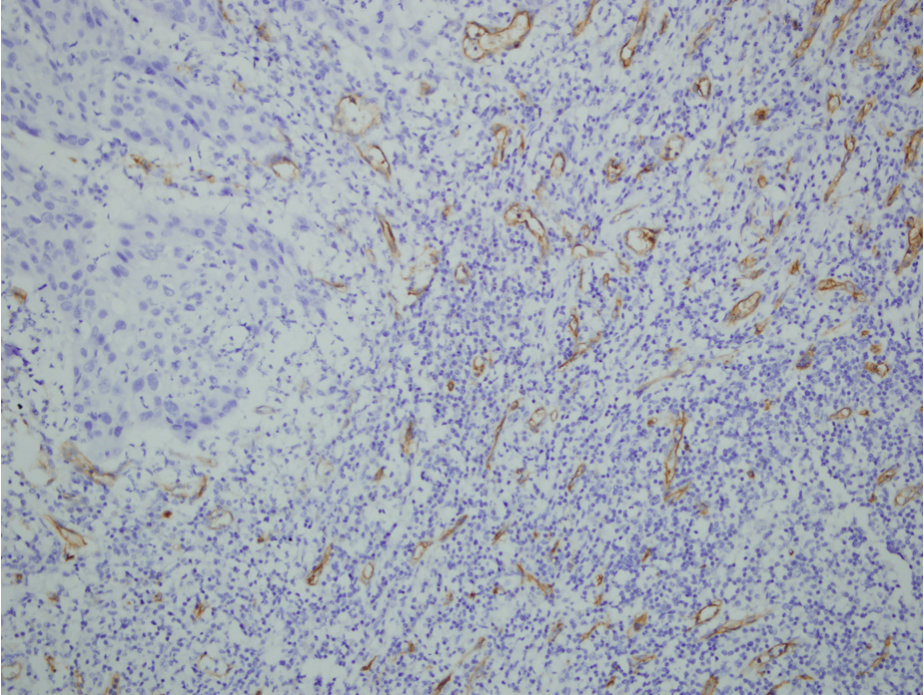
**Immunohistochemical demonstration of microvessels with anti-CD34 in grade 2 laryngeal squamous cell carcinoma, (density of microvessel counted as 43) ×200**.

*p: grade 1 vs 3 = 0.007; grade 2 vs 3 = 0.04; MVD, microvessel density; PTEN, phosphatase and tensin homolog deleted in chromosome ten; VEGF, vascular endothelial growth factor;

The mean number of vessels (mean ± SEM) was 43.0 ± 2.6 in 76 cases with grade 1, 40.4 ± 2.8 in 41 cases with grade 2, and 59.5 ± 5.8 in 23 cases with grade 3 (Figure 3). The mean number of vessels was significantly different in different grades (p = 0.003). Although there was no significant difference between grade 1 and grade 2 in terms of the number of vessels (p = 0.825), there was a significant difference between grade 1 and grade 3 and between grade 2 and grade 3 (p = 0.007 and p = 0.04, respectively; Table 1). There was no significant relationship between PTEN and VEGF expression and the mean number of vessels (p = 0.120 and p = 0.175, respectively) (Table 2).

**Table 2:** Association of PTEN and VEGF expression and pathological features of laryngeal carcinoma (mean ± standard error of mean).

		PTEN				VEGF			
					
	N(%)	Negative 26	Mild 43	Severe 71	p	Negative 10	Mild 28	Severe 102	p
MVD	140	51.9 ± 5.9	46.7 ± 3.6	41.3 ± 2.3	*0.120*	32.0 ± 6.3	44.4 ± 4.4	46.4 ± 2.3	*0.175*
Tumor size (mm)		3.1 ± 0.2	2.8 ± 0.2	3.2 ± 0.1	*0.311*	1.9 ± 0.2	3.1 ± 0.2	3.1 ± 0.1	*0.0006*
Lymph node					*0.024*				0.048*
No	91	18(20)	34(37)	39(43)		10(11)	20(22)	61(67)	
Yes	49	7(14)	9(19)	33(67)		0	8(16)	41(84)	
Cartilage invasion					*0.128*				0.129*
No	69	15(22)	25(36)	29(42)		10(14)	13(19)	46(67)	
Yes	71	11(16)	18(25)	42(59)		0	15(21)	56(79)	

*: Mild and negative cells were combined in VEGF line because of less than 5 in some cells (Fisher's exact test). MVD, microvessel density; PTEN, phosphatase and tensin homolog deleted in chromosome ten; VEGF, vascular endothelial growth factor.

While there was no significant relationship between PTEN expression and tumor size and cartilage invasion (p = 0.311 p = 0.128), there was a significant relationship between PTEN expression and lymph node metastasis (p = 0.024, chi-square = 7.479). There was a significant relationship between the severity of VEGF expression and tumor size (p = 0.006), lymph node metastasis (p = 0.048) but not cartilage invasion (p = 0.129) (Table 2).

There was no significant relationship between MVD, lymph node metastasis, and cartilage invasion (p = 0.815, p = 0.204). There was also no significant relationship between PTEN and VEGF expression (p = 0.161).

## Discussion

Angiogenesis is an essential process in the growth of the primary tumor and the development of metastasis [1]. VEGF plays a key role in tumor angiogenesis and it has been identified in many malignancies, including head and neck squamous cell carcinoma [2,7,12,13]. However, a number of different factors can regulate VEGF expression including hypoxia, cytokines, oncogenes and tumor suppressor genes [14]. PTEN, a tumor suppressor gene, is a PI3K antagonist. PI3K has a role in many cellular events such as cell proliferation and survival, protein synthesis and tumor growth. PI3K and Akt have a role in tumor growth and angiogenesis by regulating VEGF and hypoxia-inducible factor-1 expression [3]. Our results showed that there was no correlation between PTEN expression and VEGF expression which has not been investigated in the previous studies of the squamous cell carcinoma of the larynx. Also there was not a significant relationship between PTEN expression and tumor grade, tumor size and cartilage invasion in the laryngeal squamous cell carcinoma. This study demonstrates that VEGF expression is closely related to the poor clinicopathologic features including tumor grade, tumor size and lymph node metastasis of laryngeal squamous cell carcinoma.

After the first demonstration of the prognostic value of tumor angiogenesis in 1988 in malignant melanoma, VEGF expression has been studied in various tumor. The relationship of VEGF expression with parameters such as tumor differentiation, lymph node metastasis, and invasion depth has been reported in various tumors [12,13,15,16]. The findings of studies assessing the relationship between VEGF expression and tumor differentiation, stage, and lymph node metastasis differ in head and neck carcinomas [13,17,18]. In a study reporting that there was no relationship between VEGF expression and tumor grade and localization in head and neck epidermoid carcinomas, it was reported that there was a correlation between increased VEGF expression and shortened life span [19]. In a series of head and neck epidermoid carcinomas with only nine cases of laryngeal carcinoma, there was no relationship between VEGF expression and histological grade and lymph node metastasis but VEGF expression was correlated with life span, especially in tumors of the larynx and the oral cavity [18]. Tae et al. [8] reported that there was no correlation between VEGF expression and tumor differentiation, stage, and localization in carcinomas of the head and neck region. They also stated that VEGF expression was at higher levels in benign lesions than in dysplasias and carcinomas, and suggested that VEGF may be effective in regulating the mucosa functions in normal physiological conditions. Unlike our study and other studies, VEGF expression was at higher levels in the group without lymph node metastasis in this study [8]. The finding in our study that there was a significant relationship between VEGF expression and tumor differentiation, size, and lymph node metastasis supports the association of increased VEGF expression with invasive and aggressive forms of laryngeal tumors. The reasons for the different findings in our study may be the differences in assessment of VEGF expression and the fact that the other studies included tumors with different localizations in the head and neck region.

The relationship between MVD and prognostic parameters in head and neck epidermoid carcinoma could not be completely explained [20]. In spite of studies finding a relationship between high MVD and tumor progression, tumor stage and grade, studies suggesting that there was no such relationship have also been reported [7,8,12,20-22]. Tse et al. [19] conducted a study on 186 cases with head and neck epidermoid carcinomas including 34 cases with laryngeal carcinoma. They reported that there was no relationship between MVD and tumor stage, grade, localization, metastasis or prognosis. It was reported in this study that VEGF was strongly expressed but there was no relationship between VEGF expression and MVD in head and neck epidermoid carcinomas. MVD assessment was performed by two different methods by selecting hot-spot and random sites based on the fact that epidermoid carcinoma was poor for stroma and had little vascular proliferation and hot-spot sites could not be selected properly. It was reported in two assessments that there was no relationship between MVD and VEGF expression and clinicopathologic parameters. Kyzas et al. [18] reported that there was no relationship between MVD and tumor grade, localization and lymph node metastasis in 69 cases with head and neck epidermoid carcinoma, and although they found a tendency of increase in MVD with the increased VEGF expression, this increase was not significant. In this study it was reported that they performed with CD31 and CD34 and that the correlation between VEGF expression and MVD was weaker when CD34 was used [18]. The increased MVD in less differentiated tumors in our study suggested that the increase in MVD may be related to loss of differentiation. There may be several reasons why we did not find a relationship between VEGF expression and MVD. VEGF expression is only one of the angiogenic factors. Many molecules such as angiogenin, interleukin 8 and 10, platelet-derived endothelial growth factor, fibroblast growth factor, and angiopoetins have a role in tumor angiogenesis [23]. Besides forming new vessel buds, VEGF is also used as an autocrine growth factor for tumor cells. The presence of VEGF receptors on tumor cells in head and neck epidermoid carcinomas has been demonstrated [8]. Besides, it is known that other cellular elements such as macrophages and fibroblasts express VEGF at various levels [23]. This point of view may explain the lack of a significant correlation between VEGF expression and MVD. Another reason for the discrepancy between VEGF expression and MVD and between MVD and the other parameters may be the lack of a direct method to demonstrate angiogenesis [24]. The conventional antibodies used to show vessels are not specific to active angiogenic vessels [19]. It has been reported that using antibodies that show endothelial cell proliferation to assess tumor vascularization would be beneficial [24]. The lack of a significant relationship between VEGF expression and MVD shows that tumor angiogenesis is a complex process [23].

In addition to extracellular signals such as growth factors, the activation of oncogenes or the mutations of tumor suppressor genes such as PTEN and p53 trigger tumor angiogenesis. PTEN mutations were reported in ovarian, brain, breast, thyroid, endometrium and stomach tumors [25-28]. The relationship between PTEN expression and tumor differentiation, lymph node metastasis, and invasion depth has been reported in tumors of the brain, prostate, breast, and stomach [26,28,29]. While the loss of PTEN expression has been found to be correlated with the loss of differentiation in tumors of the oral cavity, it has also been reported that there was no significant relationship between PTEN expression and the tumor stage, differentiation, lymph node metastasis in laryngeal carcinoma [30]. Squarize et al. [31] found a negative relationship between PTEN expression and the grade in oral squamous cell carcinomas and reported that PTEN was an independent prognostic factor. In a study reporting that there was a decrease in PTEN expression in laryngeal tumors, there was no relationship between PTEN expression and tumor localization, tumor size, differentiation and stage [32]. We could not find a correlation between PTEN expression and tumor size, differentiation, lymph node metastasis and invasion depth. The relationship between PTEN expression and lymph node metastasis was the opposite of the expected; therefore, further studies are required on this topic.

The relationship between PTEN and angiogenesis has been explained by PTEN being a PI3K antagonist and therefore inhibiting angiogenesis [3]. It has been reported that the decrease in PTEN expression was correlated with VEGF expression and MVD increase in gastric and colon tumors [16,33]. Chung et al. [34] reported in their study with invasive ductal carcinoma that there was no relationship between PTEN expression and VEGF expression and MVD. Similarly we were not found significantly correlation between PTEN and VEGF expression and MVD. Further studies are needed to clearly explain the relationship between PTEN expression and angiogenesis.

In conclusion, VEGF expression was found to be correlated with grade, tumor size and lymph node metastasis in laryngeal tumors. However there was no correlation between PTEN expression and poor prognostic clinicopathologic factors. Increased VEGF expression may be useful to choose patients who are candidates for anti-VEGF treatment [35]. The lack of significant association between PTEN expression, VEGF expression and MVD indicates that to reveal the complex phenomenon of tumor angiogenesis in the laryngeal squamous cell carcinoma further investigation is needed.

## Competing interests

The authors declare that they have no competing interests.

## Authors' contributions

YS and FK participated in conception and design. YS wrote the initial version of the manuscript and participated in data collection and analysis. SG, SA, FK, BK performed data analysis and edited the manuscript. All authors read and approved the final version of the manuscript.
